# Rapid Microbiological Diagnostics for Sepsis: Narrative Review of Current and Prospective Approaches

**DOI:** 10.1097/CCE.0000000000001415

**Published:** 2026-05-27

**Authors:** Luke B. Harrison, Zahra N. Sohani, David Lasry, Mathew P. Cheng, Todd C. Lee, Ahmed Babiker, Sameer S. Kadri, Alexander Lawandi

**Affiliations:** 1 Division of Infectious Diseases, Department of Medicine, McGill University Health Center, Montréal, QC, Canada.; 2 Division of Medical Microbiology, Department of Laboratory Medicine, McGill University Health Center, Montréal, QC, Canada.; 3 Division of Infectious Diseases, Department of Medicine, Rush University Medical Center, Chicago, IL.; 4 Critical Care Medicine Department, NIH Clinical Center, Bethesda, MD.; 5 Critical Care Medicine Department, National Institutes of Health Clinical Center, Bethesda, MD.; 6 Critical Care Medicine Branch, National Heart Lung and Blood Institute, Bethesda, MD.; 7 Department of Critical Care Medicine, McGill University Health Centre, Montréal, QC, Canada.

**Keywords:** antimicrobial susceptibility testing, blood culture, metagenomic sequencing, molecular diagnostics, rapid diagnostics, sepsis

## Abstract

**OBJECTIVES:**

In this review, we aim to provide critical care clinicians with a concise introduction to the current and prospective tools that exist for rapid diagnostics in sepsis employed in the microbiology laboratory. Our objective is to provide a primer for clinicians to engage with their colleagues in the microbiology laboratory for the selection and implementation of new and emerging tools.

**DATA SOURCES:**

The primary literature, restricted to peer-reviewed sources, was queried using relevant search terms (e.g., sepsis, rapid diagnostics, microbiology, etc) using PubMed and Google Scholar (until February 2025), as well as review of citations of relevant articles.

**STUDY SELECTION:**

After initial searches, literature was screened by each author responsible for the sections of this review: blood culture-based methods (L.B.H.), nonblood culture-based molecular diagnostics (D.L.), and antigen-based methods (Z.N.S.). Titles and abstracts of individual articles were reviewed by the respective section authors and articles describing microbiological diagnostic techniques that decrease the turnaround time for the identification of microorganisms and/or antimicrobial susceptibility testing with relevance to the diagnosis of sepsis were retained.

**DATA EXTRACTION:**

Data from individual studies was extracted by each respective section author using Zotero reference management software and synthesized narratively.

**DATA SYNTHESIS:**

Rapid diagnostics for sepsis can be broadly divided into three categories: those applied to incubated positive blood culture specimens, and culture-independent approaches applied directly to clinical specimens, which can be further divided into those based on the direct detection of the nucleic acids of microorganisms, and those based on the detection of antigens. Blood culture-based approaches rely on biological amplification of microorganisms present but aim to measure this amplified signal directly to speed identification of microorganisms or antimicrobial resistance relative to traditional plate-culture-based workflows. Nucleic acid and antigen detection methods can be performed directly on clinical specimens, and so promise more rapid diagnostics in sepsis, but with method-specific tradeoffs in sensitivity, specificity, and interpretation.

**CONCLUSIONS:**

Evolutionary refinements of blood culture-based diagnostic approaches have decreased time to actionable information significantly while emerging and established culture-independent approaches can reduce time to actionable information to a few hours. In aggregate these interventions may have important clinical benefits, yet significant heterogeneity exists in the applicability and availability of technologies.

**KEY POINTS**

This narrative review examines technologies for rapid microbiological diagnostics in sepsis and is meant to be a primer for critical care physicians. Blood cultures remain the gold standard tests in the diagnosis of sepsis, and evolutionary progress and new technologies are reducing the turnaround time to actionable results. In parallel, culture-independent methods based on the detection of the nucleic acids or antigens of infecting microorganisms promise actionable information within hours directly from clinical specimens. Both approaches are reducing turnaround time in sepsis diagnostics, but the performance characteristics and applicability of technologies remain heterogeneous.

Sepsis is a life-threatening condition resulting from an immune response to infection and is associated with a mortality rate on the order of 20–30% (1, 2). Early administration of appropriate antimicrobial therapy results in a mortality benefit for patients with septic shock (3–7). The benefit is likely to be greatest for sepsis caused by bacterial organisms and supports the early initiation of broad-spectrum antimicrobial therapy in septic shock (8). However, the value of empiric therapy depends on its activity against the infecting pathogen, and the rise in multidrug-resistant organisms threatens this in many epidemiological contexts (9–11). Furthermore, recent evidence has suggested the potential for harm by overly broad antibiotics (12).

Although the epidemiology of sepsis varies with the clinical and geographic context of the critical care unit, sepsis in association with a bacterial infection is most common (13). There is no universal assay for the diagnosis of the microbiological etiology of sepsis, but bacterial cultures, particularly of the blood, remain cornerstones in obtaining an etiological diagnosis (6). Importantly over the last 20–30 years, significant scientific and technological effort (14) on both culture-dependent (15) and culture-independent approaches (16) have been made to more rapidly provide actionable diagnostic information on the microbial cause of sepsis to clinicians. Critical care physicians, dealing with populations at high risk of mortality, must be familiar with these advances to ensure their appropriate utilization. Here, we review existing and prospective approaches to speed the microbial diagnostics in sepsis, to provide a concise introduction to the current tools that exist for diagnostics in sepsis beyond conventional culture. First, we will focus on blood cultures as a key modality, summarizing selected current and prospective technologies to decrease the turnaround time (TAT) for identification and susceptibility testing results from positive blood cultures. Second, we will discuss existing and emerging culture-independent technologies for the diagnosis of sepsis directly from patient specimens.

## BLOOD CULTURE-BASED APPROACHES

**Figure 1.**
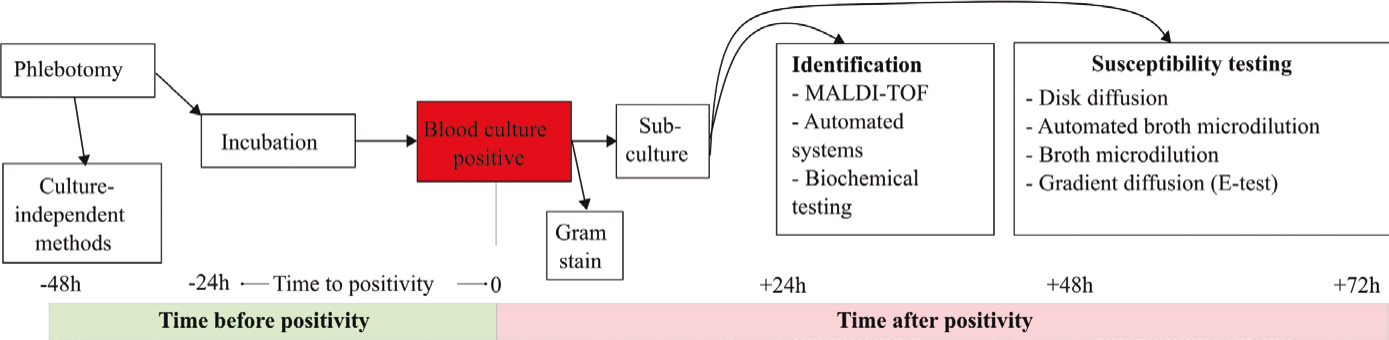
Standard blood culture workflow and approximate timelines for microbial identification and antimicrobial susceptibility testing. MALDI-TOF = Matrix-Assisted Laser Desorption/Ionization Time-of-Flight.

Culture of blood for the identification of pathogens has been the gold standard test for diagnosis of bacteremia and candidemias for over a century (17, 18). Continuous refinement over the last 50 years in blood culture media, additives (e.g., antibiotic binding agents, bacterial growth supplements), and incubators have resulted in optimized, semiautomated blood culture systems (**Fig. 1**) (15, 19). When growth is detected by the blood culture incubator, the bottle is said to be “positive,” and this defines the time to positivity (TTP). A Gram stain is then performed, and a series of primary agar plates are then inoculated from the positive bottles to isolate bacterial colonies for further identification and antimicrobial susceptibility testing (AST). The median TTP is approximately 12 hours of incubation, but can occur in as few as 5–6 hours in the case of a high-grade bacteremia, and is also dependent on the culture system and the organism’s growth rate (20–23). It is critical to ensure that cultures are collected before the initiation of broad-spectrum antimicrobials and that sufficient blood volume is collected (24). A “traditional” blood culture system yields an initial Gram stain result within an hour after positivity, final identification 24–48 hours after positivity and final AST in approximately 48–72 hours (15). Numerous improvements have been developed to speed up various aspects of the blood culture laboratory workflow (15), including both microorganism identification and AST, and we review selected techniques below. Evidence for the clinical benefit, including mortality benefit, of these rapid diagnostic tests has been analyzed in systematic reviews, but this evidence is conditioned on associated interventions that help interpret and apply the results, primarily antimicrobial stewardship programs (25, 26). **Supplemental Table 1** (https://links.lww.com/CCX/B626) summarizes important and commonly used blood culture-based systems.

### Techniques to Decrease Time to Identification of the Microorganism

#### Direct From Positive Blood Culture Bottles

Exploiting the success of Matrix-Assisted Laser Desorption/Ionization Time-of-Flight (MALDI-TOF) Mass Spectrometry-based microbial identification systems, which identify bacteria through top-down proteomics measured by mass spectrometry from colonies growing on primary plates, protocols have been developed to apply MALDI-TOF directly to positive blood culture bottles (27). This is a rapid technique that can yield results within several hours (28). One such implementation, the Rapid MBT Sepsityper (Bruker, Billerica, MA) has been shown to correctly identify 86% of monomicrobial positive blood cultures to the species level in real-world conditions, with comparatively better performance on typical pathogens (29). Disadvantages of these approaches include unreliable performance in mixed blood cultures, which can occur, for example, in intra-abdominal sepsis, and the need for confirmation with agar-plate-based culture. When applied to bloodstream infection, a large randomized controlled trial of implementation of MALDI-TOF on positive blood cultures reduced TAT to identification from 56.5 to 38.5 hours but did not demonstrate mortality benefit (30). Blood culture workflows are relatively low-cost per test compared with newer rapid platforms, but downstream costs are driven by length of stay, escalation of antibiotics, and contamination. Implementation of MALDI-TOF has substantial capital cost as the instruments are expensive, but they have very low per-isolate cost (reported estimates around ~$0.50/identification in routine practice [31]), making it financially attractive when specimen/patient volume is sufficient.

Nucleic acid amplification-based tests (NAATs) can be similarly directly applied to positive blood cultures. In the last decade, rapid multiplex polymerase chain reaction (PCR) assays (e.g., BioMérieux BioFire FilmArray BloodID and Blood Culture Identification 2 [BCID2] [BioMérieux, Salt Lake City, UT] [32], Cobas Eplex System [33] with the BCID Gram-positive [GP], Gram-negative, and fungal pathogen panels [Roche Diagnostics, Basel, Switzerland])) have been developed and are able to identify a defined subset of common pathogens in several hours. As an example, the BloodID and BCID2 panels include 33 and 43 pathogens, respectively, and 10 antibiotic resistance genes (see below), claim favorable performance characteristics, rapid TAT of typically several hours, and there is evidence of reduction in the time to prescription of appropriate antimicrobials (34). The main disadvantage to multiplex PCR assays is their cost and the fact they are limited to the detection of prespecified organisms (although these contain the most commonly encountered pathogens in the septic patient [12, 35]) and resistance mechanisms included in the panel. These tests are typically “high per test/high clinical leverage.” However, published cost-effectiveness modeling for BioFire BCID-type testing suggests that even with meaningful test costs, reductions in length of stay can make them cost-effective or cost-saving in many settings, suggesting careful consideration warranted beyond that of the platforms themselves (36).

An alternative approach to identification based on nucleic acids is microarray technology, which uses assays composed of arrays of short nucleic acid probes specific to DNA and RNA targets in organisms of interest. An example of this approach is Diasorin Verigene GP and Gram-negative tests (Diasorin, Saluggia, Italy) (37). Although the underlying technology differs, the microarray approach shares similar advantages and disadvantages to multiplex PCR tests, and demonstrates similar TATs. Another set of technologies employs an automated fluorescent in situ hybridization (FISH) technique (FISH). FISH conducted with peptide nucleic acid probes can be used to directly bind and detect pathogen DNA in specimens. One such implementation, the Accelerate Pheno system uses a set of 16 hybridization probes specific to the genus or species level to identify organisms in positive blood culture bottles (38). A final set of hypothesis-free techniques applied to positive blood culture bottles is both targeted or shotgun metagenomic sequencing (these technologies are explained in greater detail in the *Approaches to Molecular Diagnostics on Nonculture Amplified Specimens* section). Broad-based (e.g., 16S ribosomal RNA [rRNA] gene) or pathogen-specific PCR assays on positive blood cultures (39) have been mostly applied as reference methods to identify the organism(s) present when no growth is obtained on subculture of a positive blood culture. Along with shotgun metagenomic sequencing of positive blood culture bottles (40), these methods are not generally intended for rapid identification, may have longer TAT, and whose clinical utility is still being defined (39).

#### Techniques From Rapid Bottle Subculture

Rapid bottle subculture can be used to isolate colonies of rapidly growing organisms. Prewarmed nutritive agar plates are inoculated from the positive blood culture bottle and immediately incubated (41). These plates are then inspected several hours later and if visible growth is present, subjected to MALDI-TOF. This short-term incubation method can decrease the TAT for preliminary identification to several hours for quickly growing bacteria (e.g., *Escherichia coli*) (42). This technique is sometimes referred to as “hot chocolate” in reference to the chocolate (lysed RBCs) agar plates used in many protocols.

### Rapid Antimicrobial Susceptibility Testing Techniques Applied Directly to Positive Blood Culture Bottle Aspirates

#### Macroscopic Growth

Although considered less reliable than standard AST methods due to the nonstandardized inoculum, validated methods for AST directly from blood culture bottle aspirates rather than isolated bacterial colonies from plate subculture are now endorsed by the Clinical and Laboratory Standards Institute (43) and the European Committee on Antimicrobial Susceptibility Testing (44). This approach can even be automated (45) and can yield preliminary AST information in as few as 4 hours. However, this requires a monomicrobial bloodstream infection with specific pathogens for which this method has been validated (e.g., *E. coli*, *Klebsiella pneumoniae*, *Klebsiella oxytoca*, *Enterobacter cloacae* complex, *Acinetobacter baumannii*, *Pseudomonas aeruginosa*, *Enterococcus faecalis*, *Enterococcus faecium*, *Staphylococcus aureus* [43]) and only for specific antibiotics.

#### Automated Growth Monitoring Techniques

Diverse biotechnologies have been developed to detect the presence and kinetics of bacterial growth in the presence of different antimicrobials to detect growth inhibition and/or metabolic or other changes indicative of susceptibility. For example, the VITEK REVEAL system uses chromatographic detection of metabolic bioproducts, and thus growth of bacteria, exposed to antimicrobials to determine susceptibility in less than 6 hours from blood culture positivity (46). The system can provide organism identification and selected antimicrobial susceptibility results within approximately 5–6 hours after blood culture positivity (47). Clinical evaluations demonstrate substantial reductions in time to actionable results compared with conventional workflows. Its principal advantages are speed, phenotypic readout, and compatibility with existing automated laboratory infrastructure. Limitations include a restricted organism and antibiotic menu, reduced performance in polymicrobial cultures, and the need for confirmatory conventional AST. As with other rapid phenotypic systems, inappropriate reliance on preliminary results without clinical and microbiological context may risk premature de-escalation or escalation in complex infections, although a small single centered study failed to show harm (46) and the concordance of the AST results between the REVEAL and standard of care are reportedly greater than 95% (47). Another example is the Accelerate Pheno system, which combines automated FISH for organism identification with real-time morphokinetic single-cell imaging to determine phenotypic antimicrobial susceptibility directly from positive blood cultures. It delivers species-level identification in approximately 90 minutes and full MIC-based susceptibility results within 6–7 hours (38), representing one of the fastest end-to-end phenotypic AST platforms currently available. Multiple clinical studies have demonstrated marked reductions in time to both effective therapy and de-escalation, with downstream effects including shorter ICU stays, reduced broad-spectrum antibiotic exposure, and improved antimicrobial stewardship performance when embedded in coordinated care pathways. Its strengths lie in rapid, true phenotypic AST and broad applicability to common bloodstream pathogens. However, the system entails high capital and per-test costs, has a finite organism-drug menu, and may fail or perform suboptimally in mixed cultures or low-burden specimens. As with all rapid platforms, clinical benefit is contingent on integration with stewardship programs and careful interpretation of early results in the context of host severity, infection source, and local epidemiology.

#### Rapid Phenotypic Techniques

A further class of rapid techniques detects the presence of resistance mechanisms directly using biochemical assays or other means. For example, immunochromatographic or chromogenic assays can be used to directly demonstrate the activity of bacterial enzymes conferring resistance (e.g., presence of beta-lactamases and related assays). These methods can be simple, relatively inexpensive, rapid (approximately 2 hr) and sensitive, with very good overall test performance (pooled sensitivity > 0.98, specificity 1.0) analysis (48). However, recognition of local molecular epidemiology of beta-lactam resistance is critical to the implementation and interpretation of these tests. For example, rapid assays such as Beta-Lacta and BL-RED had excellent operating characteristics to detect Ambler class A beta-lactamases (e.g., KPCs) but poor for class B and C enzymes (49). Similarly, low level beta-lactamase production may not be detected and when combined with porin mutation can still cause resistance to β-lactamases. Accordingly, these assays should be interpreted as rule-in tests for specific enzymatic mechanisms rather than comprehensive screens for β-lactam resistance. Mass spectrometry (e.g., MALDI-TOF) can also be employed to directly search for profiles suggesting the presence of resistance mechanisms, and recent bacterial fingerprint detected during species identification (50–52). Furthermore, MALDI-TOF Mass Spectrometry identification relies on matching the observed peptide/protein mass fingerprint to entries in reference spectral libraries. Because only proteins that generate characteristic peaks in the typical MALDI-TOF m/z range (often ribosomal proteins and abundant housekeeping proteins) are represented, evolving resistance enzymes that differ in sequence and expression level may not produce consistent or distinct spectral features, limiting MALDI-TOF’s ability to directly identify resistance mechanisms based on standard identification alone (53).

#### Nucleic Acid Amplification-Based Techniques for AST

Where the genetic mechanism of resistance is well characterized, PCR-based assays may be used to search for antimicrobial resistance genes in aspirates of positive blood culture bottles. For example, in blood cultures positive for GP cocci on Gram stain, specific PCR for the *mecA* gene (which mediates methicillin resistance in methicillin-resistant *S. aureus*) can be performed to rapidly identify methicillin-resistant *Staphylococcal* bloodstream infection (54). Multiplex panels exist to simultaneously examine for the presence of multiple resistance genes: for example, the BioFire FilmArray BloodID panel includes *mecA*, *vanA/B*, and *bla*KPC genes, and the BCID2 panel includes those as well as *CTX-M* (ESBL-conferring), *mcr-1* (colistin resistance-conferring), and an additional four carbapenemase genes. These assays can provide specific AST information typically within hours after blood culture positivity, allowing for a rapid tailoring of treatment of patients with bacteremic sepsis. A caveat is that phenotypic assays remain the gold standard, and occasionally discordance can be observed between genotypic and phenotypic tests, and the interpretation of discordant results is not always straightforward (55).

In practice, these platforms reduce the time to selected resistance information from 24 to 48 hours after bottle positivity with conventional workflows to approximately 1–3 hours after Gram stain, often within the same laboratory shift. In a multicenter quasi-experimental study of the BioFire BCID panel, a median reduction in time to optimal antimicrobial therapy of 18–24 hours was found when rapid molecular identification and resistance gene detection were implemented but only when paired with a stewardship program (25). More recent real-world evaluations of the BCID2 panel report actionable resistance results within 2–3 hours of positivity, enabling same-day escalation for *mecA*- or *vanA/B*-positive infections and earlier carbapenem sparing when carbapenemase genes were not detected (34).

However, these assays probe for only a finite set of predefined and well characterized targets. They cannot detect resistance mechanisms arising from altered gene expression, permeability defects, or uncommon β-lactamases, nor can they exclude resistance when a gene is absent. As a result, molecular AST panels function as early, directional tools for a narrow set of high-impact resistance determinants rather than replacements for comprehensive phenotypic AST. Their principal clinical value lies in accelerating escalation or de-escalation decisions in the first hours after blood culture positivity.

## APPROACHES TO MOLECULAR DIAGNOSTICS ON NONCULTURE AMPLIFIED SPECIMENS

Techniques to detect the presence of infecting microorganisms directly, by identifying their nucleic acids or antigens directly from patients’ specimens without culture, can significantly reduce the time needed to provide actionable information in the diagnosis of sepsis (**Supplemental Table 2**, https://links.lww.com/CCX/B626).

### Nucleic Acid Amplification Tests

NAAT can be used to detect bacteria, mycobacteria, DNA and RNA viruses, fungi, protozoa, and helminths by amplifying and identifying their nucleic acids. NAATs can be applied to wide range of sterile and nonsterile clinical specimens. The primary diagnostic uses of NAAT come in the forms of singleplex PCRs, syndromic/multiplex PCR panels, can be combined with amplicon sequencing and are an increasingly important adjunct to traditional culture-based microbiology diagnostic testing. Compared with culture, NAATs can detect organisms with greater speed (within hours) and occasionally greater sensitivity and specificity; parameters that are paramount in sepsis (56). Its sensitivity is much less impacted by antimicrobials given it does not require viable microorganisms for nucleic acid detection (57). Molecular technologies have also facilitated the detection of bacteria that are fastidious (e.g., *Neisseria*, *Campylobacter*, *Mycoplasma*) or impossible to culture on agars and broths (e.g., *Rickettsia*) (58) as well as viruses, which have traditionally been diagnosed using serological assays (59).

Singleplex PCRs are designed to detect a single microbial target. The reaction involves only one set of nucleic acid primers and probes that hybridize at conserved genetic sequences flanking the target. Clinical uses include the identification of microorganisms (e.g., severe acute respiratory syndrome coronavirus 2 or influenza or *Legionella* identification in respiratory specimens, *Clostridium difficile* toxin genes in stools). In contrast, syndromic panels leverage multiplex PCR technology. These tests use many sets of probes (anywhere from 2 to over 20) in order to identify a broad range of different microorganisms within a single clinical specimen. Typically, they are designed to amplify the most common etiologic pathogens in a given clinical syndrome (60). The Roche SeptiFast PCR system (Roche Diagnostics, Basel, Switzerland) (61) has been studied for rapid microbial identification in the blood of septic patients. This system can detect 16 bacteria, five *Candida* species, and *Aspergillus fumigatus* within 6 hours. It reports a detection threshold of 3–100 CFU/mL (depending on the microorganism) and was found to have a sensitivity of 68% (CI, 63–73%) and specificity of 86% (CI, 84–89%) compared with blood cultures based on a meta-analysis of 41 studies (61). Similar panels also exist for respiratory specimens (62, 63), stool (64), synovial fluid (65), and others. For example, the BioFire FilmArray Pneumonia panel tests for 18 common respiratory genes (including bacteria and viruses), as well as commonly encountered antimicrobial resistance genes, and has excellent operating characteristics when compared with conventional culture as the gold standard (66). However, the concordance rates vary as a function of the pathogen targeted. In ventilated ICU populations, detection of multiple targets and semiquantitative signals may reflect colonization or nonviable nucleic acid; results require correlation with clinical syndrome, imaging, and quantitative culture. In general, multiplexing allows the simultaneous search and detection of multiple pathogens while minimizing reagent, machine and technologist cost. However, as mentioned above, the operating characteristics will vary across the pathogens targeted, and this is an important consideration when considering a negative result.

One particular system that intensivists should be familiar with is the T2Candida system. The T2Candida assay is a distinct class of culture-independent assay and NAAT that uses a proprietary T2 magnetic resonance to detect amplicons that have magnetically agglomerated by the proprietary beads. The test identifies the five most common *Candida* species causing candidemia (*Candida albicans*/*Candida tropicalis*, *Candida glabrata*/*Candida krusei*, and *Candida parapsilosis*) with a reported limit of detection of ~1 CFU/mL and a TAT of 3–5 hours (67). Unlike conventional NAATs, T2Candida does not rely on prior culture or free circulating DNA and is less affected by antecedent antifungal therapy. Clinical studies demonstrate substantially earlier detection compared with blood cultures, with sensitivities in the range of 90% and specificities exceeding 95% in high-risk populations (68). In the ICU, this platform offers the potential for earlier initiation or de-escalation of antifungal therapy in suspected fungal sepsis, addressing a major diagnostic gap in candidemia. From a cost perspective; however, it is important to note that the T2Candida requires dedicated instrumentation and relatively high consumable costs. Economic analyses emphasize restricting its use to high-risk settings including the ICU and hematology populations where candidemia prevalence is sufficient to justify the costs. Cost-effectiveness/budget-impact analyses support economic plausibility under those assumptions (69). Before its implementation, therefore, a careful cost analysis should be undertaken.

Broad-range bacterial PCR sequencing offers an alternative to target-specific PCR testing. Using a PCR assay with universal primers targeting a highly conserved genetic sequence that is present in all bacterial pathogens, the bacterial 16S rRNA is amplified. The resulting amplicon, which contains both highly conserved but also sufficient variability to distinguish most bacteria to at least the genus level, is then sequenced and compared with a database of known sequences to identify the microorganism(s) present (70). A similar approach can be employed to test for eukaryotic pathogens (e.g., fungi and protozoa) using the gene encoding for the 18S or 28S ribosomal subunits (71). Commercially available products, such as Molzym’s SepsiTest (Molzym, Bremen, Germany) (72), have found utility in several clinical settings, particularly in cases of culture-negative patients with a high pretest probability of infection. This includes cardiac valve tissue for culture-negative infective endocarditis, musculoskeletal and synovial specimens, and other sterile clinical samples (73). SepsiTest was studied in patients with suspected sepsis in ICU and hematology-oncology settings: Sensitivity (compared with blood cultures) ranged from 33% to 87%, and specificity was 82.9–85.2%, and NPV was 84.7–97% (74). A similar study conducted on ICU patients with sepsis showed a higher bacterial detection rate with 16S compared with blood culture: 58% vs. 16%. The majority of the bacteria detected by 16S but not culture were clinically adjudicated as being causative in the septic presentation (75). The above studies suggest 16S may add sensitivity in detecting microbial etiologies of what would otherwise be labeled “culture-negative sepsis.”

NAATs have several limitations. False positive (FP) results can diminish test specificity, there are several causes of FP NAAT testing, but among the most concerning is cross-contamination of samples during processing. False negatives, diminishing sensitivity, can also be encountered due to low DNA concentration owing to the lack of culture amplification or due to analytical factors, such as ineffective DNA extraction from cells, the use of paraffin-embedded samples, and/or failure to remove sample-based compounds that can interfere with the reactions, called inhibitors (76). The high sensitivity of many of these assays allows the detection of very small microbial burdens without clear thresholds for what might be “clinically significant” or diagnostic. For instance, *C. difficile* toxin may be detected by PCR in stool specimens of entirely asymptomatic patients without clinical colitis, which may represent colonization (77), or a patient with demonstrated bacterial sepsis may test positive for a respiratory virus. Regarding broad range bacterial PCR, it should be noted that the SepsiTest is not currently commercially available in the United States, and its use is therefore geographically restricted. Furthermore, while the underlying assay can be completed within hours, the TAT is often substantially longer in practice due to the need for sequencing. These tests are frequently performed in reference or specialized laboratories, require manual processing, sequencing, and expert interpretation, and commonly yield results on the order of 24–72 hours after specimen receipt. In applications such as explanted cardiac valve tissue, the test is therefore best viewed as a high-yield adjunct to conventional microbiology rather than a bedside or real-time diagnostic for acute management. Its principal value lies in resolving otherwise culture-negative infections and guiding definitive therapy, rather than in altering antimicrobial decisions within the first hours of sepsis management.

Rather than targeting specific genes or regions for amplification, shotgun metagenomic sequencing, a process by which all nucleic acids are extracted and sequenced directly, can be applied to clinical specimens (78). This yields thousands to millions of sequencing reads. Computational approaches are then used to exclude host (e.g., human) reads and assign each nonhost read a taxonomic identity using a library of known microbial genomes (79). These approaches, and in particular the bioinformatic methods, are not currently standardized nor trivial (70, 80). Nevertheless, shotgun metagenomic approaches are being explored in sepsis using primarily blood along with other clinical specimens (81, 82). Karius Laboratory (Karius Inc., Redwood City, CA) produced a blood/plasma test that can identify microbial DNA of over 1200 target organisms, including bacteria, fungi, protozoa, and viruses. This test is also referred to as a “liquid biopsy” as it can detect cell-free DNA from deep-seeded tissue infections that “spills” into the blood (83, 84). Metagenomic methods offer the promise of rapid, hypothesis-free identification of infecting pathogens directly from clinical specimens, including mixed infections and even the possibility of genotypic resistance prediction. However, this promise is tempered by concerns over the lack of standardization of methods, relatively high cost (approximately U.S.$2000 per test [85], although exact pricing may vary), and the interpretation of results. Although in theory, metagenomic sequencing can be conducted quickly (86), in practice, delays in shipping, batching, and reporting can lead to a TAT of several days if the test is not conducted at the ordering institution, which takes the test out of the realm of rapid diagnostics.

### Antigen-Based Techniques

Antigen tests are a nonculture-based method that detect antigens shed from the pathogen of interest (87, 88). Their utility compared with traditional methods is predicated on simplicity, rapidity, their noninvasive nature, and that they remain unaffected by prior antibiotic administration, as well as a potentially longer half-life allowing for prolonged detection (88). **Supplemental Table 3** (https://links.lww.com/CCX/B626) highlights the most commonly encountered antigens and noteworthy clinical and microbiological characteristics, with further details included in the **Supplemental Material** (https://links.lww.com/CCX/B626).

## CONCLUSIONS

Sepsis is a life-threatening syndrome of immune activation in response to microbial infection with a high rate of critical care admission and mortality. Accurate and timely provision of antimicrobial therapy is key to outcomes and underscores the importance of rapid diagnostics. Blood cultures remain the cornerstone of sepsis diagnostics, and existing and emerging technologies are incrementally reducing TAT to deliver clinically actionable information. Antigen-based techniques have an important adjunctive role in certain sepsis syndromes. Finally, while they will likely dominate rapid diagnostics for sepsis in the medium to long term, culture-independent molecular techniques, including metagenomic sequencing and NAAT-based techniques, remain important adjuncts to traditional culture-based microbiological diagnostic testing.

## Supplementary Material


