# Supplementary material for: Rapid Microbiological Diagnostics for Sepsis: Narrative Review of Current and Prospective Approaches
Source: Crit Care Explor. 2026 May 27;8(6):e1415. doi: 10.1097/CCE.0000000000001415 (PMC13215682; doi:10.1097/CCE.0000000000001415)
Supplement: Supplementary file 1 [file cc9-8-e1415-s001.pdf]

Supplemental Material- Antigen-Based Techniques

Pneumococcal infections. The diagnosis of pneumococcal infections usually relies on cultures of blood, sputum, and CSF, which can have poor sensitivity and delay diagnosis by up to 3-5 days[92]. By contrast, pneumococcal urine antigen test (UAT) detects the presence of the C-polysaccharide antigen common to all serotypes of *S. pneumoniae* in the urine of patients[92]. A meta-analysis of over 12,300 patients (~1,550 with confirmed pneumococcal pneumonia) reported pooled sensitivity of approximately 66% (95% CI: 62–69%) and specificity of 90% (95% CI: 85–93%)[94]. Although results are available rapidly, it is unclear whether pathogen-directed therapy as a result of UAT portends benefit for the patient[95,96]; prospective cohorts and randomized trials have demonstrated potential impacts on antimicrobial stewardship, but data on clinical outcomes is inconsistent[93,97].

Legionnaires' disease. Similarly, urine antigen tests can be used for the diagnosis of pneumonia caused by *Legionella pneumophila*. *Legionella* pneumonia is responsible for around 4% of community-acquired pneumonia and accounts for 2% to 15% of patients as the cause of severe CAP requiring hospitalization, including in the ICU[98,99]. Legionnaire's disease has a mortality rate of between 5-30% in hospitalized patients and close to 50% among patients admitted to the ICU[100]. While routine culture of *Legionella* pneumonia is possible, it is technically challenging due to the bacterium's specific requirement for nutrients and slow growth. *L. pneumophila* urinary antigen (UAT) detects *L. pneumophila* serogroup 1, which accounts for between 50% and 70% of cases of *Legionella* pneumonia[101, has] sensitivities between 87% and 97%, while specificities range from 86–100%[102.] Negative tests do not exclude infection and do not provide information on Legionnaire's disease caused by non-pneumophila serogroup 1. While empiric therapy for CAP

includes coverage for pneumococcal infections, Legionnaires' disease is not consistently empirically treated[103.] Several studies report reduced ICU admissions and mortality with reflex Legionella UAT[104,105], n Others maintain no benefit[101,106.]

Aspergillosis. Fungal pneumonia is often a differential diagnosis considered in immunocompromised patients, and can be frequently seen in the patients with prolonged mechanical ventilation[107]. Invasive pulmonary aspergillosis (IPA) is mainly caused by *Aspergillus fumigatus*, where the fungus can invade the tracheeobronchial tree and lung resulting in a spectrum of illness from inflammatory granuloma formation to necrotizing pneumonia[108,109]. Consensus guidelines for invasive fungal infections in critically ill hosts lacking traditional risk factors have been proposed to guide diagnosis[110]. *Aspergillus* galactomannan, frequently referred to simply as galactomannan (GM), a polysaccharide antigen that exists in the cell walls of *Aspergillus* species, is used to facilitate diagnosis[108]. GM may be released into the blood even in the early stages of *Aspergillus* invasion, and the presence of this antigen can be sustained for several weeks[108]. Sensitivity and specificity can vary greatly based on the type of specimen (BAL vs. serum), cut-off values used to signify a positive result, and whether the patient is neutropenic; most studies find sensitivities of approximately 70% and specificities of 90%[108,111–113.] A negative BAL galactomannan result substantially reduces the likelihood of invasive pulmonary aspergillosis. However, a positive BAL GM will not always discriminate between colonization and invasive disease and patients' characteristics and radiographic features are imperative in making a diagnosis and subsequently initiating treatment[114.]

Histoplasmosis, blastomycosis, and cryptococcosis. Important fungal infections leading to severe illness requiring interface with critical care include pulmonary, CNS, or disseminated infections caused by *Histoplasma capsulatum, Blastomyces dermatitidis,* and *Cryptococcus spp. Histoplasma and Blastomyces* are dimorphic fungi endemic to Ohio and Mississippi River Valleys, the Great Lakes region, St. Lawrence River Valley, and the southeastern United States, and are variably present in other regions globally[115]. Clinical presentation can include a self-limited pulmonary infection to severe pulmonary disease and disseminated disease, an entity of concern among patients with impaired cellular immunity, including those with AIDS, recent solid organ transplantation, and patients with hematological malignancies[116]. Blastomycosis, notably, can occur in immunocompetent individuals[117]. Similarly, infections caused by *Cryptococcus*, a yeast, often involves the CNS or the lungs, but disseminated disease can affect any organ, and can be present in both immunocompromised and immunocompetent individuals. Mortality, particularly from cryptococcal meningoencephalitis is high, ranging between 24 to 47%[118]. In 2022, the WHO listed *Cryptococcus* as a fungal pathogen of priority[119]. Detailed information on antigen tests in diagnosis are presented in Table 3.

Beta D-Glucan. A cell wall component found in many fungal species, the detection of beta-D-glucan (BDG) can aid in diagnosing fungal infections, particularly those caused by *Candida*, *Aspergillus*, and *Pneumocystis*. However, major limitations are that it cannot be used to monitor therapy, and its lack of specificity; BDG can be falsely elevated with use of certain antibiotics[120], recent IVIG or albumin administration, with dialysis, and with some bacterial infections, such as *S. pneumoniae* and *Pseudomonas aeruginosa*[121]. These limitations significantly impair its utility in the ICU setting. Additionally, some fungal species either do not produce BDG or do so at low

levels and therefore cannot be used to identify important infections caused by *Cryptococcus* or Zygomycetes[122]. Despite these limitations, some studies have shown that BDG testing may support antifungal stewardship in the ICU and can reduce costs[123], but without impact on patient outcomes[124]. CandiSep, a multicentre trial, reported reduced antifungal exposure in the intervention arm, however, it did not affect mortality[125]. Similarly, an observational ICU cohort shows that BDG negative results have been associated with earlier de-escalation or discontinuation of empiric antifungal treatment without increased risk of missed fungal infections[126].

Antigen-based assays are among the least resource-intensive rapid diagnostics, requiring minimal capital investment and modest laboratory expertise as many are done on the benchtop. However, their economic value is tightly linked to clinical context. While individual tests are inexpensive relative to molecular platforms, indiscriminate use can erode cost-effectiveness through low diagnostic yield and false-positive driving downstream investigations or therapy and resulting prolonged ICU and hospital admissions. For example, a European analysis estimated the all-in cost of pneumococcal urinary antigen testing at approximately €25–30 per assay, with relatively little direct impact on patient care[127]. In fungal diagnostics, the cost signal is dominated not by the assay itself but by its influence on antifungal prescribing: β-D-glucan and galactomannan testing derive value primarily by enabling earlier cessation or avoidance of empiric therapy but then the ability to provide a rapid turnaround becomes paramount[123]. Galactomannan testing carries costs on the order of $100–150 per test in North American settings (which could be shouldered by the patients)[128], whereas cryptococcal antigen lateral-flow assays remain exceptionally inexpensive—often only a few dollars per test—facilitating broad implementation where disease prevalence and clinical impact justify screening[129].

Supplemental Table 1. Selected blood culture-dependent tests and adjuncts to speed microbiological identification and antimicrobial susceptibility testing in sepsis

Technique	Sample	Principle	Examples	Major Limitations	Turn Around Time[a]
**Approaches to speed identification of positive blood cultures**					
Mass spectrometry (MALDI-TOF)	Bottle aspirate	Identification of microorganisms by proteomic comparison to reference profiles	Rapid MBT Sepsityper	Unreliable if multiple organisms present<br>Requires confirmation with routine methods<br>Increased lab work load	Hours
	Rapid subculture				Hours
NAAT	Bottle aspirate	Identification of organisms by amplification of nucleic acids using targeted primers and probes	BioFire FilmArray BloodID, BCID2<br>Cobas Eplex System (BCID-GN, BCID-GP, BCID-FP)	Limited to pre-defined pathogens	Minutes to hours
DNA microarray	Bottle aspirate	Identification of organisms by amplification of nucleic acids using targeted oligonucleotide probes	Diasorin Verigene Gram-positive and Gram-negative tests	Limited to pre-defined pathogens	Hours
fluorescent in-situ hybridization technique (FISH)	Bottle aspirate	Identification of organisms by fluorescent in situ hybridization of species-specific probes	Accelerate Pheno system (AXDX)	Limited to pre-defined pathogens	Minutes to hours

Metagenomic sequencing	Bottle aspirate	Identification of a pathogen(s) through the extraction and complete sequencing of nucleic acids and in silico pathogen identification		Difficult interpretation of significance of low abundance microbial reads<br>Complex computational methods required	Days
**Approaches to speed antimicrobial susceptibility testing (AST) of positive blood cultures**					
Standard methods (disk diffusion / broth microdilution)	Bottle aspirate	Application of standard AST methods to aspirates of positive blood cultures rather than defined inoculum from primary plates	EUCAST RAST	Unreliable if multiple organisms present<br>Validated for a limited number of organisms	Hours
Automated growth monitoring	Bottle aspirates	Application of diverse technologies to detect growth kinetics or morphological changes in bacteria in the presence of antimicrobials to determine susceptibility	VITEK REVEAL<br>Accelerate PhenoSystem	Limited to pre-defined pathogens<br>Unreliable if multiple organisms present	Hours
NAAT	Bottle aspirate	Nucleic acid amplification methods to directly detect genetic resistance determinants	BioFire FilmArray BloodID	Limited to pre-defined pathogens and AST targets<br>Unreliable if multiple organisms present	Minutes to hours

Rapid phenotypic methods	Bottle aspirate	Multiple methods: immunochromatographic, chromogenic, mass spectrometry (MALDI-TOF) to directly detect resistance determinants	Carba-NP	Limited to pre-defined enzymatic targets and/or mechanisms of resistance	Minutes to hours

[a] Time from blood culture positivity. The times provided here are illustrative, and will vary depending on many local (both laboratory and health system) factors

Supplemental Table 2. Culture independent rapid microbiological identification methods in sepsis based on the direct detection and/or sequencing of nucleic acids

Molecular Technique	Description	Examples	Major limitations	Turn Around Time[a]
**Nucleic acid amplification (NAAT) tests**				
Singleplex PCR	Designed to detect and/or quantify a single microbial species through a nucleic acid probe that hybridizes to a conserved genetic sequence.	*Clostridioides difficile* toxin PCR	Limited to single target	Minutes to hours
Syndromic panels (multiplexing)	Uses many sets of probes in order to identify a broad range of different microorganisms within a single clinical specimen. They are designed to amplify the most common etiologic pathogens in a given clinical syndrome.	SeptiFast PCR system (Roche)	Limited to pre-defined pathogens	Minutes to hours
Broad-range bacterial PCR sequencing (BRBPS)	This approach employs a universal probe that hybridizes with a highly conserved genetic sequence that is present in all bacterial pathogens, the gene encoding the 16S ribosomal subunit RNA. After conventional PCR amplification, the amplicon is sequenced and then compared to a database of known sequences to produce a genus and/or species result.	SepsiTest (Molzym)	DNA from contaminant organisms may be amplified and identified	Hours to days
**Whole genome sequencing**				
Shotgun metagenomic sequencing	Identification of a pathogen(s) through the extraction and complete sequencing of nucleic acids in a patient specimen	Cell-Free DNA Liquid Biopsy	Difficult interpretation of significance of low	Hours to days

		(Karius test)	abundance microbial reads Complex computational methods required	

[a] Time from blood culture positivity. the times provided here are illustrative, and will vary depending on many local (both laboratory and health system) factor

Supplemental Table 3. Culture independent rapid microbiological identification methods in sepsis based on the direct detection of microorganism antigens

Antigen	Target	Body fluid	Sensitivity/ Specificity	Important cross-reactions	Turn Around Time[a]	Primary Clinical Role
**Pneumococcal**	C-polysaccharide antigen of *Streptococcus pneumoniae*	Urine	Sensitivity 70-80% and Specificity: 90%	Recent pneumococcal vaccinations; other *Streptococcus species* (e.g. *constellatus)*	Minutes to hours, generally performed in hospital laboratories	Non invasive diagnosis of pneumococcal disease (bacteremia, pneumonia, meningitis)
Legionella pneumophila	Lipopolysaccharide in the cell wall of *L. pneumophil*a serogroup 1	Urine	Sensitivity: 87%-97% and Specificity: 86–100%	None consistently reported	Minutes to hours, generally performed in hospital laboratories	Non invasive diagnosis of legionnaire’s disease, severe Community acquired
**Galactomannan**	Cell walls of *Aspergillus* species	Serum BAL	Sensitivity: 70% and Specificity: 90%	*Penicillium, Alternaria, Paecilomyces, Fusarium, Histoplasma* and *Blastomyces*	Days, generally performed in larger hospitals or reference laboratories.	Identification of invasive pulpmary aspergillosis
Histoplasma	*Histoplasma capsulatum* capsular antigens (mostly	Serum* Urine	Sensitivity: 80-90%	*Blastomycosis, Paracoccidioid*	Days to weeks, generally	Diagnosis of acute and

	galactomannan)†	BAL<br>CSF	and specificity: 100%	*omycosis*, *Penicilliosis*	performed in a reference laboratory	subacute histoplasmosis, typically pulmonary but can be disseminated
Blastomyces	*Blastomyces dermatitidis* antigen (galactomannan)[c]	Serum<br>Urine[b]<br>BAL<br>CSF	Sensitivity: 95.00% and specificity: 100% with a cutoff of 0.31 ng/mL.	*Candida tropicalis, Coccidioides immitis, Coccidioides posadasii, Histoplasma capsulatum* and *duboisii, Paracoccidioides brasiliensis, Talaromyces marneffei* and *Aspergillus nidulans* **Antigen has near cross reactivity with histoplasma antigen**	Days to weeks, generally performed in a reference laboratory	Diagnosis of acute and subacute blastomycosis, typically pulmonary but can be disseminated
Cryptococcus spp	Latex agglutination against capsular polysaccharide antigens of *Cryptococcus neoformans*	Serum<br>CSF		*Trichosporon beigelii*	Hours, generally performed in hospital laboratories	Cryptococcal meningitis and pneumonia

[a] Illustrative turnaround time for each assay along with general information concerning which laboratories perform these tests. However, this will vary depending on local, regional, and national organization of laboratory services.

[b]preferred specimen for testing

[c]Histoplasma and Blastomyces antigens mostly consist of cell wall galactomannans, however in practice ‘galactomannan’ typically refers to Aspergillus-derived galactomannans

References

92. Song JY, Eun BW, Nahm MH. Diagnosis of Pneumococcal Pneumonia: Current Pitfalls and the Way Forward. *Infect Chemother*. 2013;45(4):351-366. doi:10.3947/ic.2013.45.4.351

93. Sordé R, Falcó V, Lowak M, et al. Current and Potential Usefulness of Pneumococcal Urinary Antigen Detection in Hospitalized Patients With Community-Acquired Pneumonia to Guide Antimicrobial Therapy. *Arch Intern Med*. 2011;171(2):166-172. doi:10.1001/archinternmed.2010.347

94. Yasuo S, Murata M, Nakagawa N, et al. Diagnostic accuracy of urinary antigen tests for pneumococcal pneumonia among patients with acute respiratory failure suspected pneumonia: a systematic review and meta-analysis. *BMJ Open*. 2022;12(8):e057216. doi:10.1136/bmjopen-2021-057216

95. Camou F, Issa N, Bessede É, Mourissoux G, Guisset O. Usefulness of pneumococcal antigen urinary testing in the intensive care unit? *Med Mal Infect*. 2015;45(8):318-323. doi:10.1016/j.medmal.2015.08.002

96. West DM, McCauley LM, Sorensen JS, Jephson AR, Dean NC. Pneumococcal urinary antigen test use in diagnosis and treatment of pneumonia in seven Utah hospitals. *ERJ Open Res*. 2016;2(4):00011-02016. doi:10.1183/23120541.00011-2016

97. Guchev IA, Yu VL, Sinopalnikov A, Klochkov OI, Kozlov RS, Stratchounski LS. Management of nonsevere pneumonia in military trainees with the urinary antigen test for Streptococcus pneumoniae: an innovative approach to targeted therapy. *Clin Infect Dis Off Publ Infect Dis Soc Am*. 2005;40(11):1608-1616. doi:10.1086/429919

98. Doebbeling BN, Wenzel RP. The epidemiology of Legionella pneumophila infections. *Semin Respir Infect*. 1987;2(4):206-221.

99. Graham FF, Finn N, White P, Hales S, Baker MG. Global Perspective of Legionella Infection in Community-Acquired Pneumonia: A Systematic Review and Meta-Analysis of Observational Studies. *Int J Environ Res Public Health*. 2022;19(3):1907. doi:10.3390/ijerph19031907

100. Andrea L, Dicpinigaitis PV, Fazzari MJ, Kapoor S. Legionella Pneumonia in the ICU: A Tertiary Care Center Experience Over 10 Years. *Crit Care Explor*. 2021;3(8):e0508. doi:10.1097/CCE.0000000000000508

101. Dionne M, Hatchette T, Forward K. Clinical utility of a Legionella pneumophila urinary antigen test in a large university teaching hospital. *Can J Infect Dis*. 2003;14(2):85-88.

102. Couturier MR, Graf EH, Griffin AT. Urine antigen tests for the diagnosis of respiratory infections: legionellosis, histoplasmosis, pneumococcal pneumonia. *Clin Lab Med*. 2014;34(2):219-236. doi:10.1016/j.cll.2014.02.002

103. Allgaier J, Lagu T, Haessler S, et al. Risk Factors, Management, and Outcomes of

Legionella Pneumonia in a Large, Nationally Representative Sample. *Chest*. 2021;159(5):1782-1792. doi:10.1016/j.chest.2020.12.013

104. Lettinga KD, Verbon A, Weverling GJ, et al. Legionnaires' disease at a Dutch flower show: prognostic factors and impact of therapy. *Emerg Infect Dis*. 2002;8(12):1448-1454. doi:10.3201/eid0812.020035

105. Endo M, Jo T, Konishi T, Kumazawa R, Matsui H, Yasunaga H. Association between the Timing of Urinary Antigen Testing and Outcomes in Legionella Pneumonia Patients: A Nationwide Database Study. *Intern Med Tokyo Jpn*. 2024;63(1):51-56. doi:10.2169/internalmedicine.1115-22

106. Falguera M, Ruiz-González A, Schoenenberger JA, et al. Prospective, randomised study to compare empirical treatment versus targeted treatment on the basis of the urine antigen results in hospitalised patients with community-acquired pneumonia. *Thorax*. 2010;65(2):101-106. doi:10.1136/thx.2009.118588

107. Pagano L, Caira M, Candoni A, et al. The epidemiology of fungal infections in patients with hematologic malignancies: the SEIFEM-2004 study. *Haematologica*. 2006;91(8):1068-1075.

108. Zhou W, Li H, Zhang Y, et al. Diagnostic Value of Galactomannan Antigen Test in Serum and Bronchoalveolar Lavage Fluid Samples from Patients with Nonneutropenic Invasive Pulmonary Aspergillosis. *J Clin Microbiol*. 2017;55(7):2153-2161. doi:10.1128/JCM.00345-17

109. Kousha M, Tadi R, Soubani AO. Pulmonary aspergillosis: a clinical review. *Eur Respir Rev*. 2011;20(121):156-174. doi:10.1183/09059180.00001011

110. Bassetti M, Giacobbe DR, Agvald-Ohman C, et al. Invasive Fungal Diseases in Adult Patients in Intensive Care Unit (FUNDICU): 2024 consensus definitions from ESGCIP, EFISG, ESICM, ECMM, MSGERC, ISAC, and ISHAM. *Intensive Care Med*. 2024;50(4):502-515. doi:10.1007/s00134-024-07341-7

111. Pfeiffer CD, Fine JP, Safdar N. Diagnosis of Invasive Aspergillosis Using a Galactomannan Assay: A Meta-Analysis. *Clin Infect Dis*. 2006;42(10):1417-1727. doi:10.1086/503427

1112. Wu Z, Wang L, Tan L, Wu J, Chen Z, Hu M. Diagnostic value of galactomannan in serum and bronchoalveolar lavage fluid for invasive pulmonary aspergillosis in non-neutropenic patients. *Diagn Microbiol Infect Dis*. 2021;99(4):115274. doi:10.1016/j.diagmicrobio.2020.115274

1113. Karapinar D. A Review of a Diagnostic Tool: Galactomannan. *J Immunol Sci*. 2018;2(5):38-42. doi:10.29245/2578-3009/2018/5.1137

114. Donnelly JP, Chen SC, Kauffman CA, et al. Revision and Update of the Consensus Definitions of Invasive Fungal Disease From the European Organization for Research and

Treatment of Cancer and the Mycoses Study Group Education and Research Consortium. *Clin Infect Dis Off Publl Infect Dis Soc Am*. 2020;71(6):1367-1376. doi:10.1093/cid/ciz1008

115. Lockhart SR, Toda M, Benedict K, Caceres DH, Litvintseva AP. Endemic and Other Dimorphic Mycoses in The Americas. *J Fungi*. 2021;7(2):151. doi:10.3390/jof7020151

116. Bradsher RW. Histoplasmosis and blastomycosis. *Clin Infect Dis Off Publ Infect Dis Soc Am*. 1996;22 Suppl 2:S102-111. doi:10.1093/clinids/22.supplement_2.s102

117. Hennessee I, Palmer S, Reik R, et al. Epidemiological and Clinical Features of a Large Blastomycosis Outbreak at a Paper Mill in Michigan. *Clin Infect Dis*. 2025;80(2):356-363. doi:10.1093/cid/ciae513

118. Bicanic T, Wood R, Meintjes G, et al. High-dose amphotericin B with flucytosine for the treatment of cryptococcal meningitis in HIV-infected patients: a randomized trial. *Clin Infect Dis Off Publ Infect Dis Soc Am*. 2008;47(1):123-130. doi:10.1086/588792

119. Chang CC, Harrison TS, Bicanic TA, et al. Global guideline for the diagnosis and management of cryptococcosis: an initiative of the ECMM and ISHAM in cooperation with the ASM. *Lancet Infect Dis*. 2024;24(8):e495-e512. doi:10.1016/S1473-3099(23)00731-4

120. Liss B, Cornely OA, Hoffmann D, Dimitriou V, Wisplinghoff H. 1,3-β-d-Glucan contamination of common antimicrobials. *J Antimicrob Chemother*. 2016;71(4):913-915. doi:10.1093/jac/dkv419

121. Wei Z, Xu J, Yuan F, et al. Pseudomonas aeruginosa infections and improper storage conditions influence the performance of 1,3-β-d-glucan in diagnosis of invasive fungal infections. *iLABMED*. 2024;2(1):53-59. doi:10.1002/ila2.35

122. Lamoth F, Akan H, Andes D, et al. Assessment of the Role of 1,3-β-d-Glucan Testing for the Diagnosis of Invasive Fungal Infections in Adults. *Clin Infect Dis Off Publ Infect Dis Soc Am*. 2021;72(Suppl 2):S102-S108. doi:10.1093/cid/ciaa1943

123. Hamilton DO, Lambe T, Howard A, et al. Can Beta-D-Glucan testing as part of the diagnostic pathway for invasive fungal infection reduce anti-fungal treatment costs? *Med Mycol*. 2022;60(5):myac034. doi:10.1093/mmy/myac034

124. Kritikos A, Poissy J, Croxatto A, Bochud PY, Pagani JL, Lamoth F. Impact of the Beta-Glucan Test on Management of Intensive Care Unit Patients at Risk for Invasive Candidiasis. Hanson KE, ed. *J Clin Microbiol*. 2020;58(6):e01996-19. doi:10.1128/JCM.01996-19

125. Bloos F, Held J, Kluge S, et al. (1 → 3)-β-d-Glucan-guided antifungal therapy in adults with sepsis: the CandiSep randomized clinical trial. *Intensive Care Med*. 2022;48(7):865-875. doi:10.1007/s00134-022-06733-x

126. Posteraro B, Tumbarello M, De Pascale G, et al. (1,3)-β-d-Glucan-based antifungal treatment in critically ill adults at high risk of candidaemia: an observational study. *J Antimicrob Chemother*. 2016;71(8):2262-2269. doi:10.1093/jac/dkw112

127. Dinh A, Duran C, Davido B, et al. Cost effectiveness of pneumococcal urinary antigen in Emergency Department: a pragmatic real-life study. *Intern Emerg Med*. 2018;13(1):69-73. doi:10.1007/s11739-016-1586-4
128. Treutel PR, Carr A, Bathina P. 735. Trends and Cost Analysis of Aspergillus Galactomannan testing at a Tertiary Medical Center. *Open Forum Infectious Diseases*. 2020;7(Supplement_1):S417-S417. doi:10.1093/ofid/ofaa439.926

129. Ramachandran A, Manabe Y, Rajasingham R, Shah M. Cost-effectiveness of CRAG-LFA screening for cryptococcal meningitis among people living with HIV in Uganda. *BMC Infect Dis*. 2017;17(1):225. doi:10.1186/s12879-017-2325-9
